# Supplementary material for: Sex-specific association of visceral adiposity index with renal dysfunction in chinese type 2 diabetes: A cross-sectional study
Source: PLoS One. 2026 Feb 25;21(2):e0343375. doi: 10.1371/journal.pone.0343375 (PMC12935224; doi:10.1371/journal.pone.0343375)
Supplement: Attachment — Submitted filename: Response to Reviewers.docx [file pone.0343375.s003.docx]

Jan 4^th^ 2026

Title: Sex-specific Association of Visceral Adiposity Index with Renal Dysfunction in Chinese Type 2 Diabetes: A Cross-sectional Study
Submission ID: PONE-D-25-64102

Dear Dr. Aleksandra Klisic,

We deeply appreciate the time and effort of the reviewers, and we thank them for their constructive assessment of our submitted manuscript. We also thank the editor for encouraging us to submit a revised version that incorporates the reviewers’ comments.We have carefully reviewed the feedback and made the necessary revisions to improve the quality and clarity of our work.

We have continued our efforts towards improving our manuscript during the review process and have made every effort to systematically address all the reviewer’s critical suggestions. As a result, and as part of our response to the reviewers’ comments, several improvements and additions have been made to the revised version of the manuscript.

We hope that these revisions satisfactorily address the concerns raised. We uploaded a copy of the revised manuscript to the *PLOS ONE* website. All authors have read and agreed to the changes incorporated in the revised version of our manuscript.

If there are any further questions, please do not hesitate to contact us.

We look forward to hearing from you at your earliest convenience.

Sincerely,

Bingyin Shi

Department of Endocrinology

The First Affiliated Hospital of Xi’an Jiaotong University

Xi’an 710061

P.R. China

E-mail: [shibingy@126.com](mailto:shibingy@126.com)

**Response to Journal Requirements:**

1. Formatting and Style:
   We have reviewed the PLOS ONE style requirements and adjusted the file naming and manuscript formatting accordingly.
2. ORCID iD:
   The corresponding author has updated their information in Editorial Manager and validated their ORCID iD as requested.
3. Ethics Statement:
   We confirm that the ethics statement is now exclusively located in the Methods section of the manuscript.
4. Data Availability:
   We have updated our Data Availability Statement. The minimal data set underlying the findings described in our manuscript has been uploaded as Supporting Information (S1 Dataset).

5. References:
We have checked the reference list for completeness and correctness, ensuring no retracted papers are cited.

**Response to Reviewer #1**

1.As much as possible it is better to avoid abbreviations on the Abstract section.
**Reply:**

Thank you for this suggestion. We have revised the Abstract to minimize abbreviations. Terms such as T2DM, eGFR, and VAI have been spelled out where appropriate to improve readability.

Changes made: [See Abstract, Page 1-2]

2.The Abstract section lacks background which summarizes the magnitude and severity of renal dysfunction among T2DM patients and the real gaps filled by this specific study.

**Reply:**

We have added a background sentence to the Abstract to contextualize the study. It now briefly highlights the impact of renal dysfunction in diabetes and the gap regarding visceral adiposity indices in the Chinese population.
 Changes made: [See Abstract, Page 1]

3.How did you calculate the sample size in accordance with your source population manually or using software? 1300 Vs 1335 is unclear.

**Reply:**

We apologize for the confusion. The sample size of 1,300 was our calculated target to ensure sufficient power for sex-stratified analysis. The final number of eligible participants enrolled was 1,335, which met and slightly exceeded this target. We have clarified this distinction in the "Sample Size Calculation" section.
 Changes made: [See Methods, "Sample Size Calculation" section, Page 5]

4.What is your outcome variable? it hasn't been well emphasized.

**Reply:**

We have revised the text to explicitly state that the primary outcome variable is renal dysfunction, defined as an eGFR < 60 mL/min/1.73 m².
 Changes made: [See Methods, "Variables" section, Page 6-7]

5.What were the limitations of your study?

**Reply:**

We have reviewed the "limitations" paragraph in the Discussion section. We explicitly acknowledge the single-center design, the cross-sectional nature preventing causal inference, and potential residual confounding. We have also added a note regarding the retrospective nature of data collection.
 Changes made: [See Discussion, Page 21]

6.How did you resolve incompleteness of records, eligibility issues for the incompletes.

**Reply:**

Participants with missing data on core variables (key visceral fat markers or renal function) were excluded from the study as per our exclusion criteria. For covariates with minimal missing data (<5%), we utilized multiple imputation to minimize bias and maximize data utility. We have clarified this in the Statistical Analysis section.
 Changes made: [See Methods, "Statistical Analysis" section, Page 7-8]

7.What was the main reason why you didn't you conduct a community based study and collect primary data?

**Reply:**

This is a valuable question. We chose a hospital-based retrospective design for two main reasons:

**Clinical Precision:** Hospital records provide more comprehensive and precise laboratory measurements (e.g., specific biochemical markers) compared to what is typically feasible in large-scale community screenings.

**Efficiency:** Utilizing electronic medical records allowed us to efficiently analyze a large cohort of confirmed T2DM patients to generate hypotheses regarding sex-specific differences, which can subsequently be tested in future prospective community-based studies.

8. Lastly, it is better to present your findings using tables and figures as well.

**Reply:**

Thank you for this suggestion. We have ensured that our findings are comprehensively presented using Tables and Figures. Regarding the placement of figures, we initially moved them into the text body per your suggestion; however, in strict adherence to the journal’s submission guidelines received from the Editorial Office, we are required to upload figures as separate high-resolution files (TIFF/EPS) rather than embedding them in the manuscript. We have ensured that the Figure Legends are clearly placed within the Results section to indicate the logical flow of the findings.

Changes made: [Figures have been uploaded as separate files per journal policy; Figure Legends are retained in the Results section]

**Response to Reviewer #2**

1.Your figures should have been in the body of the results and not the appendix to make the work easier to understand. While the tables are okay in the appendix, the figures should be in the body of your work.

**Reply:**

We fully appreciate and agree with the reviewer’s view that placing figures within the text improves readability. However, we have received specific technical instructions from the Editorial Office requiring us to remove embedded images from the manuscript file and upload them as separate standard image files (TIFF/EPS). Therefore, to comply with the journal’s production requirements, we have uploaded the figures separately. The submission system will automatically compile these files into the final PDF for your review. We have placed the Figure Legends within the body of the Results section to assist with the reading flow.

Changes made: [Embedded figures were removed and uploaded as separate files to comply with editorial instructions]

2.Instead of the meaning of all the abbreviations for indices being under table 2 in the appendix. It should preferably be in a list form and very bold so it can be properly seen. preferably just after the disclosure statement.

**Reply:**

We have created a bolded List of Abbreviations and placed it immediately after the Disclosure statement as requested, ensuring all indices are clearly defined for the reader.
 Changes made: [See Page 23]
