# Supplementary material for: Sex-specific association of visceral adiposity index with renal dysfunction in chinese type 2 diabetes: A cross-sectional study
Source: PLoS One. 2026 Feb 25;21(2):e0343375. doi: 10.1371/journal.pone.0343375 (PMC12935224; doi:10.1371/journal.pone.0343375)
Supplement: Attachment — Submitted filename: Response_to_Reviewers_auresp_2.docx [file pone.0343375.s004.docx]

Jan 22^th^ 2026

Title: Sex-specific Association of Visceral Adiposity Index with Renal Dysfunction in Chinese Type 2 Diabetes: A Cross-sectional Study
Submission ID: PONE-D-25-64102R1

Dear Dr. Aleksandra Klisic,

Thank you for the opportunity to revise our manuscript. We are pleased that reviewer found the manuscript technically sound and well-written. The reviewer's sole comment concerns the placement of figures and figure legends; however, as explained in our detailed response below, this format is required by PLOS ONE's submission system, and therefore no changes have been made to the manuscript content.

We look forward to the publication of our manuscript in PLOS ONE.

Sincerely,

Bingyin Shi

Department of Endocrinology

The First Affiliated Hospital of Xi’an Jiaotong University

Xi’an 710061

P.R. China

E-mail: [shibingy@126.com](mailto:shibingy@126.com)

**Response to Reviewer #2**

We sincerely thank the reviewer for this thoughtful comment and fully understand the concern regarding the placement of figure legends and footnotes.

However, we would like to clarify that this format is in accordance with PLOS ONE's submission requirements. During our previous revision submission, when we included figures alongside their legends in the main text (following the reviewer's preference), the submission failed the journal's format check. We received the following specific instruction from the journal: "Please remove your figures from within your manuscript file, leaving only the individual TIFF/EPS image files. These will be automatically included in the reviewer's PDF."

Following this guidance, we removed all embedded figures and uploaded them as separate image files. Therefore, the current format—with figure legends in the manuscript and figures as separate files—is in compliance with PLOS ONE's submission guidelines. Once the manuscript is accepted and published, the figures and their corresponding legends will be properly integrated in the final published version.

We sincerely appreciate the reviewer's understanding on this matter.
